# Identification and evaluation of suitable reference genes for RT-qPCR analyses in *Trichoderma atroviride* under varying light conditions

**DOI:** 10.1186/s40694-023-00167-w

**Published:** 2023-10-03

**Authors:** Daniel Flatschacher, Alexander Eschlböck, Susanne Zeilinger

**Affiliations:** Department of Microbiology, University of Innsbruck, Technikerstraße 25, 6020 Innsbruck, Austria

**Keywords:** *Trichoderma atroviride*, RT-qPCR, Reference genes, Photobiology

## Abstract

**Background:**

*Trichoderma atroviride* is a competitive soil-borne mycoparasitic fungus with extensive applications as a biocontrol agent in plant protection. Despite its importance and application potential, reference genes for RT-qPCR analysis in *T. atroviride* have not been evaluated. Light exerts profound effects on physiology, such as growth, conidiation, secondary metabolism, and stress response in *T. atroviride*, as well as in other fungi. In this study, we aimed to address this gap by identifying stable reference genes for RT-qPCR experiments in *T. atroviride* under different light conditions, thereby enhancing accurate and reliable gene expression analysis in this model mycoparasite. We measured and compared candidate reference genes using commonly applied statistical algorithms.

**Results:**

Under cyclic light–dark cultivation conditions, *tbp* and *rho* were identified as the most stably expressed genes, while *act1*, *fis1*, *btl*, and *sar1* were found to be the least stable. Similar stability rankings were obtained for cultures grown under complete darkness, with *tef1* and *vma1* emerging as the most stable genes and *act1*, *rho*, *fis1*, and *btl* as the least stable genes. Combining the data from both cultivation conditions, *gapdh* and *vma1* were identified as the most stable reference genes, while *sar1* and *fis1* were the least stable. The selection of different reference genes had a significant impact on the calculation of relative gene expression, as demonstrated by the expression patterns of target genes *pks4* and *lox1*.

**Conclusion:**

The data emphasize the importance of validating reference genes for different cultivation conditions in fungi to ensure accurate interpretation of gene expression data.

**Supplementary Information:**

The online version contains supplementary material available at 10.1186/s40694-023-00167-w.

## Background

Light serves as a crucial environmental cue regulating many cellular processes in filamentous fungi, including growth, development, and metabolism [1]. The ability of organisms to integrate external signals, such as light, contributes to their competitiveness in the environment and constitutes an important driving force of evolution and adaptation [2]. In ascomycetes, responses to light often involve the modulation of characteristic fungal processes, including sporulation, hyphal branching, and the production of secondary metabolites and hydrolytic enzymes [1]. From a transcriptomic point of view, light regulates the expression of numerous genes in various fungi [3–5]. Members of the genus *Trichoderma* are highly competitive soil-borne filamentous fungi and are widely used in agricultural and biotechnological applications, including biocontrol, plant growth promotion, and production of industrial enzymes. *Trichoderma atroviride*, in particular, is commonly used as a biocontrol agent because of its ability to parasitize and compete against a variety of phytopathogenic fungi [6]. Light profoundly influences the physiology of *Trichoderma*, affecting growth, conidiation, secondary metabolism, stress response, as well as mycoparasitic activity [7–10].

Quantitative reverse transcription PCR (RT-qPCR) is a commonly used technique for measuring gene expression levels in a wide range of biological samples. One of the key advantages of RT-qPCR is its high sensitivity and specificity, which allows the detection of even small changes in transcript levels [11]. However, accurate and reliable quantification of gene expression in RT-qPCR experiments requires appropriate normalization of the data to account for differences in RNA quantity, quality, and enzymatic efficiency [12]. Normalization of RT-qPCR data is typically achieved by using reference genes, which are genes that are stably expressed under different experimental conditions and are used to normalize the expression levels of target genes [13]. The use of reference genes is crucial for accurate and reliable gene expression analysis, as it allows the identification of true changes in gene expression levels that are not confounded by experimental variations [14].

Commonly used reference genes for RT-qPCR studies in filamentous fungi are associated with basic cellular structures (e.g. tubulin and actin) and genes involved in basic cellular processes, such as glycolysis (glyceraldehyde-3-phosphate dehydrogenase (*gapdh*)), synthesis of ribosomal subunits (rRNA), electron transport, and protein degradation (ubiquitin) [15–18]. These genes have been widely used as reference genes due to their assumed stable expression across different experimental conditions. However, the suitability of these reference genes can be limited under certain conditions and across different fungal species [19–21]. The study of gene expression in filamentous fungi is essential for understanding their physiology, development, and interactions with the environment. Yet, identifying stable reference genes for RT-qPCR analysis poses challenges, due to the complex growth morphology and variable gene expression patterns observed in filamentous fungi. In recent years, there has been growing awareness of the importance of appropriate reference gene selection in RT-qPCR experiments, and several statistical algorithms, such as geNorm [22], NormFinder [23], and BestKeeper [24], have been developed to assess reference gene stability across different samples and experimental conditions.

While Brunner et al. [25] have identified and validated reference genes in *T. atroviride* under conditions such as growth on different carbon sources, development, and confrontation, no investigation has focused specifically on different light conditions in this species. In this context, our study aimed to identify stable reference genes for RT-qPCR based gene expression analysis in *T. atroviride* grown under two different light conditions, thereby enhancing the accuracy and reliability of transcription analysis in this important model organism. A set of 10 candidate reference genes was evaluated in fungal cultures incubated either in the presence of a white light–dark cycle or complete darkness. Five in silico algorithms including RefFinder [26], GeNorm [22], NormFinder [23], BestKeeper [24] and comparative $\Delta$Ct [27] were used to evaluate the expression stability of each candidate gene, and the relative stability level under representative conditions was obtained. In addition, the transcript levels of two genes, a polyketide synthase gene (*pks4*) and the lipoxygenase-encoding gene *lox1*, both known to be involved in light-response in *Trichoderma* [28, 29], were analyzed to demonstrate the effectiveness and reliability of the selected reference genes.

## Results

### Primer performance analysis

Ten genes were selected as candidate reference genes for *T. atroviride* P1. The amplification efficiencies, correlation coefficients ($\text {R}^2$) and slope values of the candidate reference genes are shown in Table 1.

**Table 1:** Amplification efficiencies, correlation coefficients ($\text {R}^2$) and slope of RT-qPCR primers of ten candidate reference genes

Candidate reference gene	Amplification efficiency	$\text {R}^2$	Slope
*act1*	97.21	0.995	− 3.1695
*btl*	104.78	1.000	− 3.2123
*fis1*	96.57	0.988	− 3.4068
*gapdh*	100.16	0.993	− 3.3180
*rho*	90.26	0.991	− 3.5798
*rpl6e*	106.08	0.990	− 3.1842
*sar1*	109.33	0.999	− 3.1168
*tbp*	106.17	0.999	− 3.1824
*tef1*	109.87	0.999	− 3.1060
*vma1*	109.76	0.997	− 3.1082

The specific fragments of all candidate reference genes were successfully amplified from *T. atroviride*-derived cDNA using the primers given in Table 2. Each PCR reaction resulted in a single product with the expected fragment size confirming the amplification specificity of the primers (data not shown). Moreover, the melting curve of each primer pair showed a single peak, further verifying the specificity of the primer pairs and ruling out the presence of primer dimers (Additional file 1). The amplicon sizes ranged from 112 to 305 bp, and the amplification efficiencies (E) of the primer pairs ranged from 90.26% (*rho*) to 109.87% (*tef1*). In addition, all 10 correlation coefficients ($\text {R}^2$) were $\ge$0.99. These results indicate that the primers designed for all 10 candidate reference genes meet the standards for RT-qPCR and could be used in subsequent experiments.

**Table 2:** List of candidate reference genes and target genes used in the RT-qPCR analysis

Gene symbol	Accession	Primer sequence (5’-3’)	Amplicon length [bp]	Tm [$^{\circ }\hbox {C}$]	Final concentration [nM]
*act1*	420323	F: AGGTAGGCGTGACTTGAACA R: TGAGCTTGGCACGAGTGATT	122	F: 58.95 R: 59.96	400
*btl*	422722	F: ATGGCTGCTTCTGACTTCCG R: TGTTCTGCACGTTTCGCATC	166	F: 60.39 R: 59.77	400
*fis1*	303068	F: TACAAGCTCGGCAACTACGG R: CGACCTTGTCGTCGATGAGT	112	F: 60.11 R: 59.83	400
*gapdh*	443719	F: CGAGGAGATTACCAACGCCA R: GTTGTTGTTGCCGAGCATGT	167	F: 59.83 R: 59.97	400
*lox1*	330921	F: GCCTTCTTACCGAAGCCA R: CTGCCAGCATCCTTGGAA	200	F: 56.97 R: 57.25	400
*pks4*	489002	F: AGCGACGACTACAGAGAGGT R: TGTCACATTCATTGCGCCAG	202	F: 60.04 R: 59.48	400
*rho*	331454	F: TCAACTTCACCAGCCCACTG R: GGTCGAGTTCGAGCAATCCT	153	F: 60.18 R: 59.83	400
*rpl6e*	414343	F: AGGCTTTCTTCAAGCAGGGA R: CGGCTGCTGTTGATCTCCT	168	F: 59.23 R: 59.78	400
sar1*	457369	F: CTCGACAATGCCGGAAAGACCA R: TTGCCAAGGATGACAAAGGGG	305	F: 63.12 R: 60.83	400
*tbp*	444185	F: GAAGACGATTGCTCTGCACG R: ATGCGAAGATGAGGGCAGTT	184	F: 59.63 R: 59.75	400
*tef1*	419042	F: CACCATCGACATTGCCCTCT R: CAGTCAGCCTGGGAAGTACC	182	F: 60.11 R: 59.75	400
*vma1*	173248	F: ACTGGATGAACTGGACCGTG R: TCTCGCTGCTTTTTGTTGGC	130	F: 59.68 R: 59.97	200

Oligo orientations (F, R), primer sequences, amplicon lengths, melting temperatures (Tm), and final primer concentrations are given

*Primer from [25]

### Expression levels of candidate reference genes

**Fig. 1:**
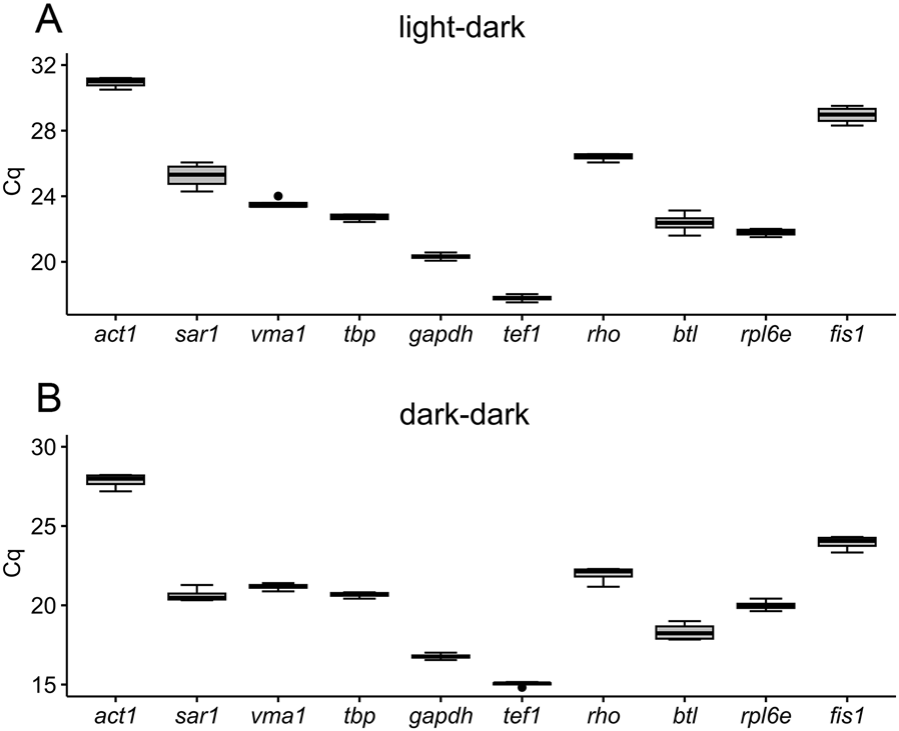
Expression variability of each of the ten tested reference gene candidates among both tested light conditions, light–dark cycle and complete darkness (dark–dark), presented as box-and-whisker plot. The line across the box indicates the median value. The box indicates the 25th and 75th percentiles, whereas the whisker represents the maximum and minimum values. **A** Raw expression of each gene in *T. atroviride* cultivated under light–dark conditions shown as Cq values. **B** Raw expression of each gene in *T. atroviride* cultivated under complete darkness shown as Cq values

Mean quantification cycle (Cq) values of the ten candidate reference genes ranged from 17.78 to 30.93 in samples derived from cyclic light–dark cultivation conditions and from 15.03 to 27.84 upon cultivation under complete darkness, showing a wide variation of expression levels among the evaluated candidate reference genes (Fig. 1). The average Cq values from all genes indicated that the two genes with the highest (*act1*) and lowest (*tef1*) expression levels are basically the same regardless of the evaluated condition.

### Expression stability of candidate reference genes

**Fig. 2:**
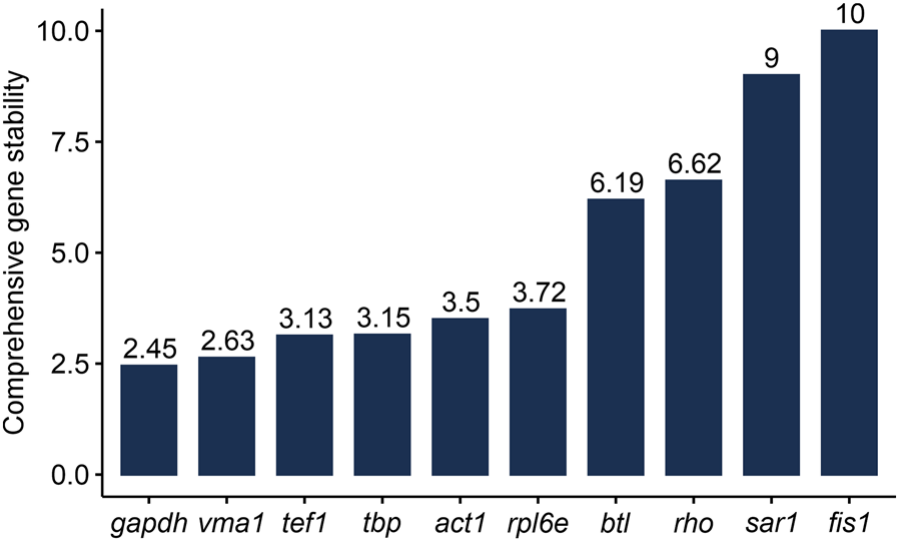
Comprehensive gene stability of the tested ten candidate reference genes over both cultivation conditions (light–dark cycle and complete darkness) as calculated by RefFinder. Lower values represent higher stability in the tested samples. See also Additional file 4

The expression stability of candidate reference genes was estimated using the geNorm [22], NormFinder [23], BestKeeper [24], comparative $\Delta$Ct [27], and RefFinder [26] algorithms. With these algorithms, reference genes can be ordered from the most stably expressed, exhibiting the lowest Cq variation, to the least stable. The stability values of the candidate reference genes observed in samples derived from light–dark cultivation (Additional file 2) indicated that *tbp* and *rho* were the most stable among the genes tested. Based on the SD values (standard deviation of the Cq value) calculated by BestKeeper, all genes were stably expressed during light–dark cultivation, as indicated by an SD of <1. However, *tef1* and *gapdh* showed the best stability values. According to the ranking by RefFinder, *tbp*, *rho*, *rpl6e*, *gapdh*, and *tef1* emerged as the most stable genes with respect to fungal cultivation under a light–dark cycle. In relation to the less stable genes, all analyzed algorithms classified *act1*, *fis1*, *btl*, and *sar1* as the least stable of all candidate genes tested. Similar to the results obtained for growth of *T. atroviride* under light–dark conditions, all tested genes were stably expressed under conditions of complete darkness, as reflected by SD values ranging from 0.11 to 0.45 (Additional file 3). The RefFinder stability classification of the tested candidate reference genes upon fungal cultivation under complete darkness indicated *tef1* and *vma1* as the most stable genes. BestKeeper identified *tef1* and *gapdh*, and GeNorm identified *tbp* and *gapdh* as the most stable candidates among the tested genes under conditions of complete darkness. *Sar1*, *btl*, and *fis1* were identified by comparative $\Delta$Ct, NormFinder and GeNorm algorithms as the least stable of all candidate genes tested. With the BestKeeper algorithm, *act1*, *rho*, and *btl* emerged as the least stable genes. The general ranking by RefFinder displayed *sar1*, *rho*, *fis1*, and *btl* as the least stable of all tested candidate genes. In order to accurately identify the most stable genes under both light conditions, all data were combined and jointly analyzed (Fig. 2, Additional file 4). NormFinder and comparative $\Delta$Ct identified *gapdh* as the most stable gene, followed by *act1* and *tef1*. GeNorm identified *vma1* and *tbp* as being most stably expressed with identical stability values (0.371). For BestKeeper, *rpl6e* displayed the greatest expression stability (SD = 0.91). The overall ranking established by RefFinder revealed that the most stable candidate reference genes were *gapdh* and *vma1*. All algorithms identified *sar1* and *fis1* as the least stable among the tested candidate genes across both cultivation conditions.

### Validation of reference gene selection

**Fig. 3:**
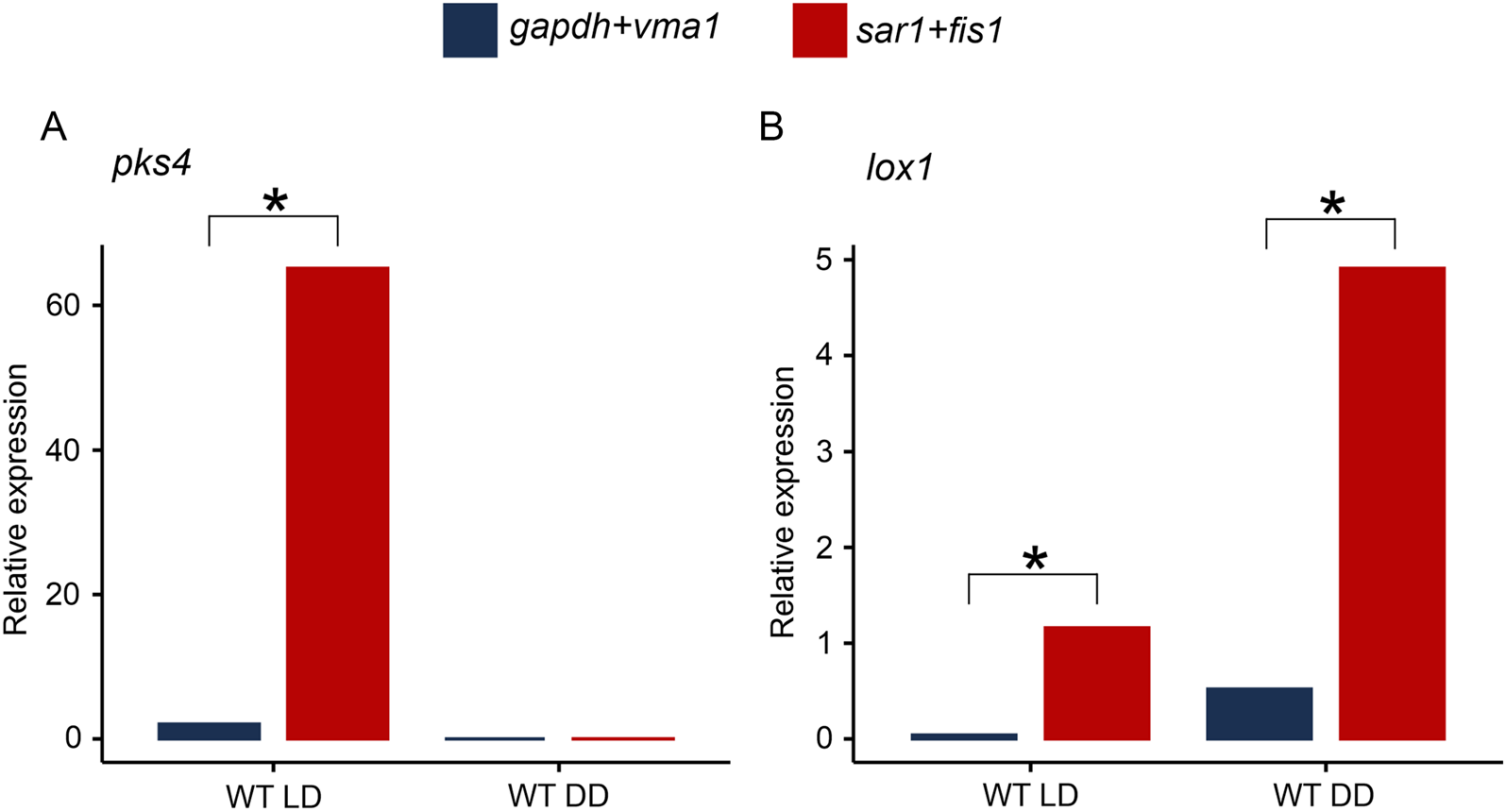
Relative expression of target genes *pks4* and *lox1* using validated reference genes including the most (*gapdh*+*vma1*) or the least (*sar1*+*fis1*) stable reference genes for normalization. **A** Relative expression of *pks4* in *T. atroviride* cultivated under light–dark conditions (WT LD) or complete darkness (WT DD) normalized to either *gapdh*+*vma1* (blue) or *sar1*+*fis1* (red). **B** Relative expression of *lox1* in *T. atroviride* cultivated under light–dark conditions (WT LD) or complete darkness (WT DD) normalized to either *gapdh*+*vma1* (blue) or *sar1*+*fis1* (red). Asterisks indicate significant differences (p < 0.05, one-way ANOVA)

The impact of selecting different reference genes on the calculation of relative gene expression was evaluated by examining the expression patterns of the target genes *pks4* and *lox1* in response to light. Two pairs of candidate reference genes, namely *gapdh* and *vma1* (the two most stable reference genes) as well as *sar1* and *fis1* (the two least stable reference genes), were employed to normalize Cq values. The obtained results revealed that *pks4* is expressed only in samples derived from cultivation under light–dark conditions, while no expression was detected upon fungal growth in complete darkness (Fig. 3A). In contrast, *lox1* expression levels were higher when *T. atroviride* was cultivated under complete darkness compared to light–dark conditions (Fig. 3B). Comparative analysis of the expression profiles demonstrated significant variation in gene expression for both target genes when different reference genes were utilized. Notably, when the most stable reference genes were employed, the obtained transcript levels for *pks4* and *lox1* under light–dark conditions were remarkably higher than when normalized with the least stable reference genes. These findings indicate a direct association between the calculated expression level of a target gene and the reference genes employed for data normalization. Inappropriate selection of reference genes, as demonstrated here for the determination of *pks4* and *lox1* gene expression, led to an overestimation of their transcript levels, underscoring the importance of validating reference genes prior to their application in gene expression studies.

## Discussion

Our findings demonstrate that *gapdh* and *vma1* were the most stable reference genes across varying light conditions for *T. atroviride*. These results are in line with previous research indicating that *vma1* and *gapdh* serve as reliable reference genes in various tissues, including other filamentous fungi such as *Neurospora crassa* [30] and *Aspergillus flavus* [31], under various cultivation conditions [32, 33]. However, numerous studies have shown that the mentioned housekeeping genes are regulated and exhibit expression variability in response to experimental conditions [34–37]. Consequently, it remains up to the researcher to judiciously select a reference gene that ensures reliable normalization within a specific experimental context. Paolacci et al. further emphasized the significance of studies aimed at identifying and validating internal control genes suitable for each unique experimental scenario, suggesting that alternative normalization genes may replace traditionally employed ones [38]. This underscores the importance of meticulous selection and validation of reference genes tailored to specific experimental conditions.

The *vma1* gene encodes the subunit A of vacuolar ATPase, which plays a critical role in regulating vacuolar pH and ion homeostasis [39]. Likewise, *gapdh* encodes glyceraldehyde-3-phosphate dehydrogenase, an essential enzyme in glycolysis [40]. Both genes are constitutively expressed and involved in fundamental cellular processes, which may explain their consistent expression patterns even across different light conditions. Consistently, *act1*, which encodes actin involved in cytoskeletal formation, demonstrated stable expression, as expected for a fundamental cellular component [41]. In *T. virens*, *T. atroviride* and *T. reesei*, the elongation factor encoding gene *tef1* maintained constant expression and was used as normalizer evaluating gene expression when these fungi were confronted with the plant pathogen *Rhizoctonia solani* [42]. Our findings also support *tef1* as a good normalizer for *T. atroviride* strain P1 grown under different light conditions, ranking after *gapdh* and *vma1*. In contrast, *fis1* and *sar1* were identified as the least stable reference genes in our study. The essential role of *fis1* in conidiation in other filamentous fungi, such as *Magnaporthe oryzae*, may explain its instable expression pattern [43]. Interestingly, a study evaluating potential reference genes in *T. reesei* found *sar1* to be the most stable gene for bioreactor cultivation, while *act1* did not rank among the most reliable reference genes [16]. In *T. atroviride*, *sar1* turned out to be the most stable reference gene for biocontrol conditions and cultivation on solid media in presence of various carbon sources, alongside *act1*. Moreover, when comparing cultivation of *T. atroviride* on solid PDA and liquid PDB, *act1* and *tef1* were identified as the most stable reference genes [25].

The choice of reference genes for normalization is crucial in gene expression studies, as it directly influences the accuracy and reliability of the results [44]. In our study, the use of *sar1* and *fis1* as reference genes led to an overestimation of transcript levels of *pks4*, encoding a polyketide synthase responsible for pigment biosynthesis in *T. reesei* [28] and *T. atroviride*, as well as *lox1*, a lipoxygenase encoding gene known to be involved in the response of *T. atroviride* to light [29]. This discrepancy demonstrates the inherent variability introduced by the selection of inappropriate reference genes and emphasizes the importance of using stable and reliable normalizers for accurate data interpretation. The overestimation of transcript levels when using unsuitable reference genes can have significant implications on the conclusions drawn and suggested biological role. It can lead to misinterpretation of gene regulation patterns, false identification of differentially expressed genes, and erroneous conclusions about the functional roles of target genes. This has previously been shown in other reports as well, where the choice of unstable genes applied for normalization led to misinterpretation of expression data [45–47].

The validation of the tested reference genes in this study also revealed an intriguing expression pattern of the examined target genes *pks4* and *lox1* in *T. atroviride* under different light conditions. Specifically, *pks4* was found to be transcribed exclusively under light–dark conditions and not under complete darkness, while *lox1* exhibited higher expression levels in complete darkness compared to light–dark conditions. The light-induced expression of *pks4* suggests its involvement in pigment biosynthesis, which is known to be influenced by the presence of light–dark cycles, consistent with the presence of pigmented spores solely in the presence of light in vivo [48]. On the other hand, the increased expression of *lox1* in complete darkness highlights its role in light-response mechanisms that are specifically triggered in the absence of light [29]. To obtain reliable and meaningful results, it is crucial to validate reference genes specific to the experimental conditions and biological system under investigation [49]. Our study emphasizes the importance of using stable reference genes like *gapdh* and *vma1* for normalization in RT-qPCR based gene expression studies in *T. atroviride* under different light conditions. The use of appropriate reference genes ensures that the expression profiles of target genes are accurately represented, enabling valid interpretations and biological conclusions.

## Conclusions

In this study, we assessed the stability of 10 candidate reference genes in *T. atroviride* upon cultivation under different light conditions using multiple evaluation algorithms. Among the examined genes, the commonly used reference gene *sar1* was identified as one of the least stable reference genes across the experimental conditions tested, along with *fis1* and *btl*. Hence, these genes are not recommended for normalization purposes in studies addressing light-dependent gene expression. In contrast, *gapdh* and *vma1* demonstrated consistent and stable transcript levels, making them suitable reference genes for RT-qPCR analysis in *T. atroviride* under varying light conditions. Therefore, we propose the simultaneous use of *gapdh* and *vma1* as optimal reference genes to enhance the accuracy and reliability of gene expression studies in *T. atroviride*, particularly when addressing light-mediated processes. We could also show that inappropriate reference gene selection led to overestimation of gene expression levels. Therefore, the validation and careful selection of reference genes are critical to ensure reliable RT-qPCR based gene expression analysis in fungi. This research provides valuable insights into the selection of stable reference genes and underscores the importance of appropriate normalization for accurate gene expression analysis, thus contributing to our understanding of gene regulation and supporting future investigations of the model mycoparasite *T. atroviride*.

## Material and methods

### Strains and growth conditions

*Trichoderma atroviride* strain P1 (ATCC 74058) was used throughout this study. For cultivation, the strain was pre-grown for three days on potato dextrose agar (PDA; Becton, Dickinson and Company, Le Pont De Claix, France) plates at 25 $^{\circ }\hbox {C}$ under either light–dark (12:12 h cycle; 1040 Lux; Snijders Micro Clima-Series TM Labs Economic Lux Chamber; Snijders Labs, Tiburg, Netherlands) conditions or in complete darkness. In order to ensure consistency of the respective cultivation condition, agar plugs (6 mm diameter) of the actively growing colony margins were propagated twice, after three days each, to the center of fresh PDA plates. For the final cultivation step, the agar plugs from the actively growing colony margins from the pre-cultures were inoculated in quadruplicates centrally on fresh PDA plates covered with a cellophane membrane. Plates were incubated at 25 $^{\circ }\hbox {C}$ under a light–dark cycle (12:12 h; 1040 Lux) or under complete darkness for 3 days. The biomass was collected, immediately frozen in liquid nitrogen, and kept at − 70 $^{\circ }\hbox {C}$ for subsequent RNA extraction.

### Total RNA extraction and cDNA synthesis

Frozen mycelia were homogenized using the CryCooler (OPS Diagnostics). Total RNA from 1 mg of each sample was extracted using TRIzol Reagent (Invitrogen, Karlsruhe, Germany). Isolated RNA was treated with DNAse I (ThermoFisher Scientific Baltic UAB, Vilnius, Lithuania) and RNA integrity and absence of genomic DNA was further assessed by electrophoresis using 1.5% agarose gels. 1 $\upmu \hbox {g}$ of treated RNA was reverse transcribed to cDNA using the Revert Aid H- First Strand cDNA Synthesis Kit (ThermoFisher Scientific Baltic UAB, Vilnius, Lithuania) with a 1:1 combination of the provided oligo(dT) and random hexamer primers. The cDNA mixture was diluted 1:10 with ddH_2_O and stored at -70 $^{\circ }\hbox {C}$ for subsequent RT-qPCR analysis.

### Reference gene selection and primer design

Ten reference genes that were commonly used in eukaryotes were selected to screen for stable gene expression in *T. atroviride* P1 grown under the described light conditions. These candidate reference genes included *sar1* (coding for a SAR/ARF type small GTPase), *act1* (actin-encoding), *vma1* (vacuolar ATPase subunit 1-encoding), *tbp* (TATA-binding protein-encoding), *gapdh* (glyceraldehyde 3-phosphate dehydrogenase-encoding), *tef1* (translation elongation factor 1a-encoding), *rho* (coding for a GTPase activator), *btl* (beta-tubulin-encoding), *rpl6e* (ribosomal protein L6e-encoding) and *fis1* (coding for a mitochondrial membrane fission protein) selected according to previously published reference gene analysis performed in *N. crassa* [30], *T. reesei* [16, 50] and *Talaromyces versatilis* [51]. Both coding and genomic sequences of each gene were obtained from the JGI *T. atroviride* P1 database (https://mycocosm.jgi.doe.gov/Triatrov1/Triatrov1.home.html). Primers for *act1*, *btl*, *fis1*, *gapdh*, *rho*, *rpl6e*, *tbp*, *tef1* and *vma1* were designed using the NCBI Primer Blast tool (https://www.ncbi.nlm.nih.gov/tools/primer-blast/), with an annealing temperature of 60$^{\circ }\hbox {C}$. Primers for *sar1* were obtained from Brunner et al. [25]. Primers were designed to span exon-exon junctions and each primer pair was separated by at least one intron to minimise the amplification of contaminant gDNA. The potential for homo- or hetero dimer formation was examined for each primer pair using the IDT Oligo Analyzer tool (https://www.idtdna.com/pages/tools/oligoanalyzer). Only primers with a $\Delta$G value greater than − 7 kcal/mole were used. Primers were obtained from Microsynth AG (Balgach, Switzerland). The specificities of the primers were verified by conventional PCR using cDNA as templates and the amplicons were visualized by electrophoresis on 2.0% agarose gels. The primer sequences are given in Table 2.

### RT-qPCR conditions and amplification efficiency

RT-qPCR was performed using the LUNA Universal qPCR Master Mix (New England BioLabs GmbH) in an $\text {qTOWER}^3$ G cycler (Analytik Jena AG, Jena, Germany). All reactions except the ones for *vma1* were performed in a 20 $\upmu \,\hbox {L}$ mixture containing 10 $\upmu \,\hbox {L}$ of Master Mix, 4 $\upmu \,\hbox {L}$ of cDNA template with final amount of 200 ng, 0.8 $\upmu \,\hbox {L}$ of each primer with final concentration of 400 nM, and 4.4 $\upmu \,\hbox {L}$ of sterile water. For *vma1*, 0.4 $\upmu \,\hbox {L}$ of each primer with a final concentration of 200 nM, and consequently 4.8 $\upmu \,\hbox {L}$ of sterile water were used per reaction. qPCR analysis included four technical replicates of each sample. Non-template controls (NTC) in which the cDNA was replaced with nuclease free water were also included for each primer pair to exclude possible contaminations. In addition, RT-minus reactions, prepared during cDNA synthesis without adding reverse transcriptase were performed to monitor for possible gDNA amplification. The qPCR cycling conditions were as follows: 95$^{\circ }\hbox {C}$ for 1 min, followed by 50 cycles of 95$^{\circ }\hbox {C}$ for 15s and 60$^{\circ }\hbox {C}$ to 65$^{\circ }\hbox {C}$ for 30s. Amplicons were verified by subsequent melting curve analysis, performed from 60$^{\circ }\hbox {C}$ to 95$^{\circ }\hbox {C}$. Amplification efficiency of each primer pair was evaluated by the standard curve method using serial dilutions of cDNA (1:10, 1:100, 1:1000, and 1:10000) as a template. The efficiencies (E) of corresponding primers were calculated in accordance with the equation $E = 1+10^{(-1/slope)}*100$.

### Stability analysis of candidate reference genes

The expression stabilities of the ten tested candidate reference genes under the two different light conditions were evaluated using RefFinder, which includes the four commonly used in silico algorithms BestKeeper [24], geNorm [22], NormFinder [23], and comparative $\Delta$Ct [27]. The candidate reference genes were ranked by each algorithm individually and RefFinder was then applied to calculate a comprehensive value based on the four individual results. The geometric means of the individual ranks were used to determine their expression stability, with the lowest value representing the most stably expressed gene while the highest representing the least stably expressed candidate.

### Validation of reference gene stability

To validate the reliability of the selected reference genes, the relative expression levels of the polyketide synthase-encoding gene *pks4* and the lipoxygenase-encoding gene *lox1* were analyzed in *T. atroviride* grown in the presence of light or darkness. The nucleotide sequences of *pks4* and *lox1* were obtained from the JGI *T. atroviride* P1 database (https://mycocosm.jgi.doe.gov/Triatrov1) and primers (Table 2) were designed using the NCBI Primer Blast tool (https://www.ncbi.nlm.nih.gov/tools/primer-blast/). The RT–qPCR conditions were set up the same as described above. The relative expression of both genes was calculated using qRAT [52] with the two most stable and two least stable candidate reference genes obtained by the comprehensive assessment used as normalizers.

### Supplementary Information

**Additional file 1: Figure S1.** Melting curves of the ten tested candidate reference genes (*sar1*, *act1*, *vma1*, *tbp*, *gapdh*, *tef1*, *rho*, *btl*, *rpl6e*, *fis1*) generated by RT-qPCR showing the specificity of the respective primers.

**Additional file 2: Table S1.** Expression stability of the tested candidate reference genes in *T. atroviride* cultivated under light–dark conditions calculated by RefFinder, GeNorm, NormFinder, BestKeeper and comparative $\Delta$Ct algorithms. Ranks are according to the value of stability of the respective algorithm.

**Additional file 3: Table S2.** Expression stability of the tested candidate reference genes in *T. atroviride* cultivated under complete darkness calculated by RefFinder, GeNorm, NormFinder, BestKeeper and comparative $\Delta$Ct algorithms. Ranks are according to the value of stability of the respective algorithm.

**Additional file 4: Table S3.** Overall expression stability of the tested candidate reference genes in *T. atroviride* cultivated under light–dark and complete darkness calculated by RefFinder, GeNorm, NormFinder, BestKeeper and comparative $\Delta$Ct algorithms. Ranks are according to the value of stability of the respective algorithm.

## Data Availability

The datasets used and/or analyzed during the current study are available from the corresponding author on reasonable request.
