# Supplementary figures and images for: Identification and evaluation of suitable reference genes for RT-qPCR analyses in Trichoderma atroviride under varying light conditions
Source: Fungal Biol Biotechnol. 2023 Oct 3;10:20. doi: 10.1186/s40694-023-00167-w (PMC10546744; doi:10.1186/s40694-023-00167-w)

*act1*

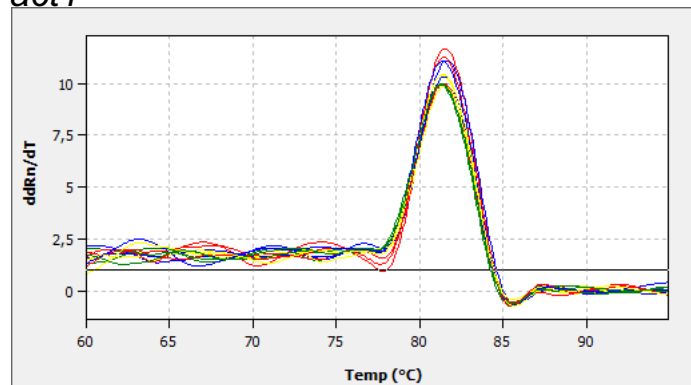

*btl*

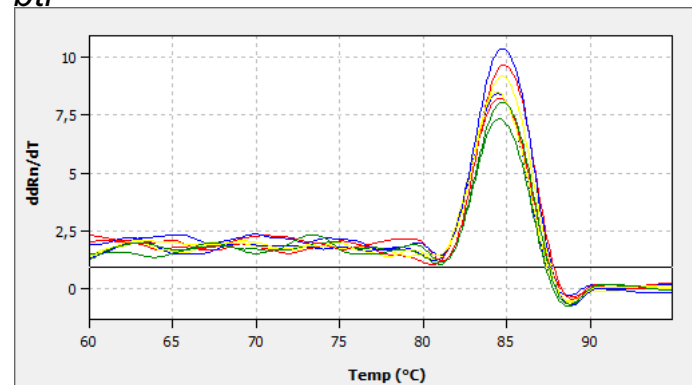

*fis1*

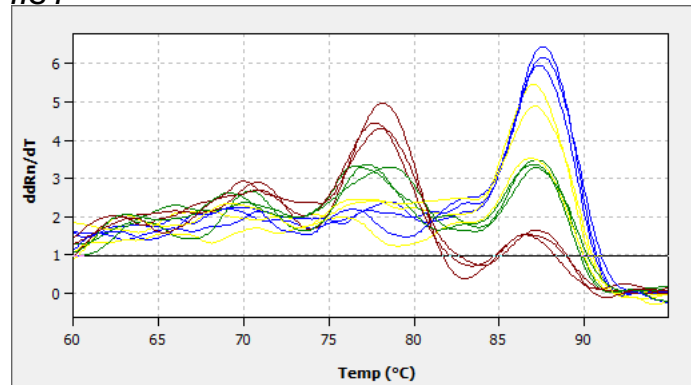

*gapdh*

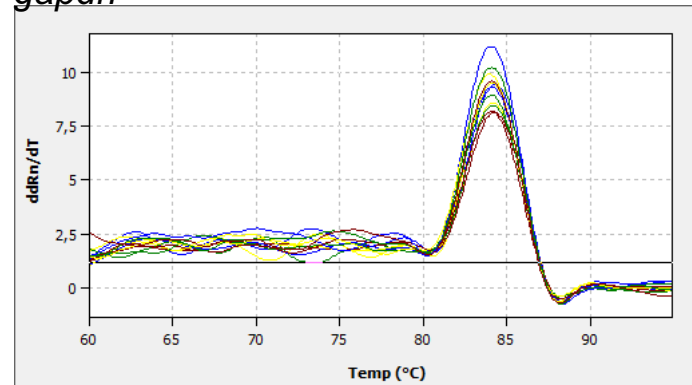

*rho*

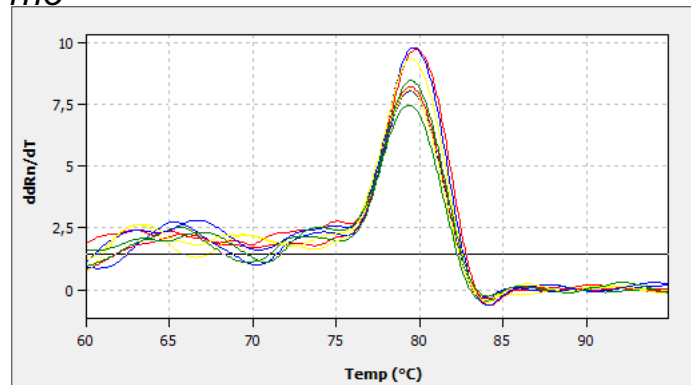

*rpl6e*

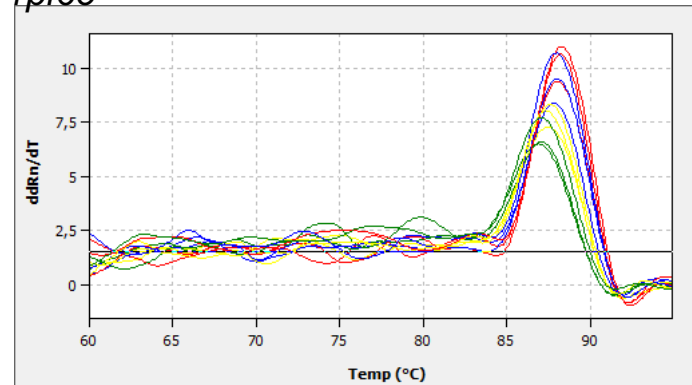

*sar1*

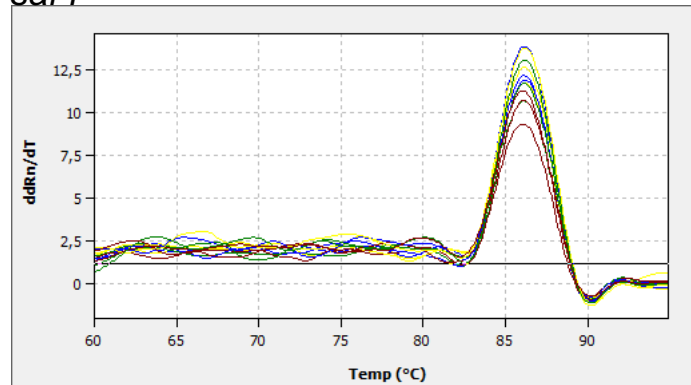

*tbp*

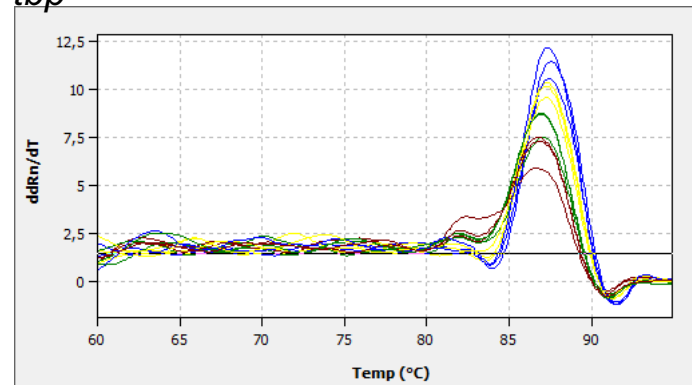

*tef1*

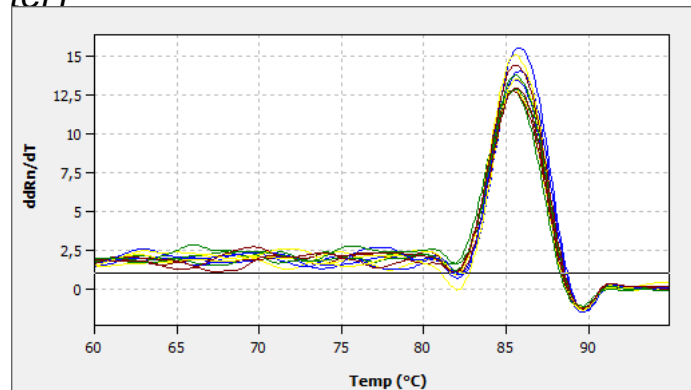

*vma1*

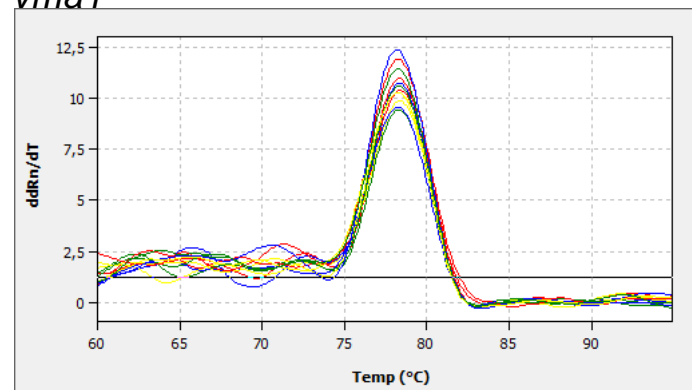

Supplement: Supplementary file 1 — Additional file 1: Figure S1. Melting curves of the ten tested candidate reference genes (sar1, act1, vma1, tbp, gapdh, tef1, rho, btl, rpl6e, fis1) generated by RT-qPCR showing the specificity of the respective primers. [file 40694_2023_167_MOESM1_ESM.pdf]
